# Supplementary material for: The Relationship between Different Amounts of Physical Exercise, Internal Inhibition, and Drug Craving in Individuals with Substance-Use Disorders
Source: Int J Environ Res Public Health. 2021 Nov 26;18(23):12436. doi: 10.3390/ijerph182312436 (PMC8656815; doi:10.3390/ijerph182312436)
Supplement: Supplementary file 1 [file ijerph-18-12436-s001.zip › ijerph-1351326-supplementary.pdf]

Internal Inhibition Scale

1. When it is wrong, I will correct it.
2. I can review and reflect on myself when others criticize.
3. I don't think it is easy for me to show good behavior.
4. I am easily discouraged by failure.
5. When I do something, I will first think about it.
6. I lose my temper easily.
7. I am often impulsive.
8. I lack confidence in myself.
9. I get upset when I do something wrong.
10. I can concentrate on doing everything.
11. I have some bad habits that can’t be changed.
12. When I am criticized for doing something wrong, I will be happy to reason with others.
13. Under the teasing of others, I can do almost everything.
14. When I encounter a problem, I like to wait and see if someone solves it.
15. I like to do something exciting when I'm bored.
16. I will do whatever it takes to beat others.
17. I often make mistakes I made before.
18. I don’t think it’s easy to concentrate on anything.
19. Sometimes I really want to throw things.
20. When I encounter difficulties, I feel helpless.
21. I often lose my confidence because of the stimulation of others.
22. When there is no supervision, I often cannot restrain my behavior.
23. In unsatisfactory situations, I am able to accept the challenge courageously.
24. When encountering setbacks, I can always find a way to solve the problem.
25. When things go wrong, my emotions often become agitated or discouraged.

The scores from low to high are (1-5): Strongly agree, Quite agree, Generally, Slightly agree, Disagree

Drug Craving Scale

1. I can't do anything without taking drugs.
2. If I stop taking drugs, my life will be depressed.
3. I don't have enough capacity to stop taking drugs.
4. I can't relax without drugs.
5. I can’t relieve my anxiety without taking drugs.
6. Taking drugs can make people forget their worries temporarily.
7. Drugs can satisfy curiosity.
8. Taking drugs will give me a stimulating feeling.
9. Taking drugs can relieve the feeling of injustice when being wronged.
10. Drug use can enhance sexual performance.
11. Drugs are a great way to refresh yourself.
12. Drugs can relieve stress.
13. I will take drugs all caused by others.
14. I only smoke once and I shouldn't be addicted.
15. As long as you don’t let others find out, it’s okay to take one or two mouthfuls.
16. I will quit the drugs after another breath.
17. The craving for drugs is a physiological response, so I can’t resist it at all.
18. If my craving for drugs cannot be satisfied, I will become more and more uncomfortable.
19. The craving for drugs drives me crazy.
20. I am always craving for drugs.
21. I will continue to crave drugs for the rest of my life.
22. I feel extremely uncomfortable physically when I cannot control my craving for drugs.
23. The thirst for drugs is far greater than one's willpower.
24. When I crave drugs, I can’t control my thoughts and ideas at all.
25. As long as I want to take drugs, I will definitely find drugs to use.

The scores from low to high are (1-5): Strongly agree, Quite agree, Generally, Slightly agree, Disagree
